# Cost–Utility Analysis of Wide-Field Imaging as an Auxiliary Technology for Retinopathy of Prematurity Care in Brazil

**DOI:** 10.3389/fped.2021.757258

**Published:** 2021-12-16

**Authors:** Luiza M Neves, Lorena M Haefeli, Andrea A Zin, Ricardo E Steffen, Zilton F. M Vasconcelos, Márcia Pinto

**Affiliations:** ^1^Instituto Nacional de Saúde da Mulher, da Criança e do Adolescente Fernandes Figueira, Fundação Oswaldo Cruz, Rio de Janeiro, Brazil; ^2^Instituto de Medicina Social, Universidade do Estado do Rio de Janeiro, Rio de Janeiro, Brazil; ^3^Departamento de Medicina, Pontifícia Universidade Católica do Rio de Janeiro, Rio de Janerio, Brazil

**Keywords:** retinopathy of prematurity, neonatal screening, diagnosis, healthcare economics, costs and cost analysis, quality-adjusted life years (QALY), telemedicine, Brazil

## Abstract

**Purpose:** To evaluate the cost–utility of wide-field imaging (WFI) as a complementary technology for retinopathy of prematurity (ROP) screening from the Brazilian Unified Health System's perspective.

**Introduction:** ROP is one of the leading causes of avoidable childhood blindness worldwide, especially in middle-income countries. The current ROP screening involves indirect binocular ophthalmoscopy (IBO) by ROP expert ophthalmologists. However, there is still insufficient ROP screening coverage. An alternative screening strategy is the combination of WFI with IBO.

**Methods:** A cost–utility analysis was performed using a deterministic decision-tree simulation model to estimate incremental cost–utility for ROP care. Two screening strategies were compared: (1) IBO and (2) combination of WFI of all eligible preterm infants and IBO for type 2 ROP or worse and for non-readable images. Eligible population included preterm infants <32 weeks of gestational age or birth weight equal to or <1,500 g. The temporal horizon was lifetime. Visual outcome data was converted to utility, and the health benefits were estimated on quality-adjusted life-years (QALY). Incremental cost per QALY gained was calculated from the health system perspective. Costs were estimated considering equipment, maintenance, consumables, and staff. A micro-costing approach was used for WFI. Two technician nurses were trained for imaging execution and had their time evaluated. Two ROP expert ophthalmologists had their time evaluated for imaging reading. One-way sensitivity analysis and probabilistic sensitivity analysis were performed.

**Results:** Combined screening strategy resulted in a cost-effective program considering 90% ROP screening coverage. Costs per examination: (1) screening with IBO: US dollar (US $) 34.36; (2) screening with combination: US $58.20; (3) laser treatment: US $642.09; (4) long-term follow-up: ranged from US $69.33 to 286.91, based on the infant's visual function. Incremental cost per QALY gained was US $1,746.99/QALY per infant screened with the combination strategy. One-way sensitivity analysis resulted in cost-effectiveness for all parameters. Probabilistic sensitivity analyses yielded a 100% probability of combination being cost-effective in a willingness-to-pay threshold of US $1,800/QALY.

**Conclusion:** The combined strategy for ROP screening was cost-effective. It enhances access for appropriate ROP care in middle-income countries and dminishes opportunity costs for ophthalmologists.

## Introduction

Retinopathy of prematurity (ROP) remains a leading cause of avoidable childhood blindness in middle-income economies, such as Brazil (1, 2). The combination of (1) high preterm birth rate, (2) improvement of neonatal care quality (leading to better survival rates), and (3) insufficient access to ROP screening and treatment are the main causes of the third ROP epidemic faced in countries such as Brazil (1, 3, 4). The effectiveness and affordability of ROP screening, diagnosis, and treatment have been well-documented (5–10).

The current screening of infants at risk of ROP, usually determined by gestational age (GA) and birth weight (BW) criteria, requires carefully timed retinal examinations by a skilled ophthalmologist. Noteworthy, fewer than 10% of infants screened for ROP in Brazil will develop type 1 disease and should be submitted to treatment within 72 h (6). However, only 52% of at-risk preterm infants are estimated to have access to ROP examinations, and probably fewer infants have access to treatment in Brazil (6, 11).

It is estimated that the Brazilian Unified Health System (SUS) provides 76% of neonatal care in the country (12). According to official data, 37,000 infants were born with BW <1,500 g in 2017 (13). Therefore, each year, ~20,000 infants would survive to 4 weeks requiring eye examinations, and ~1,600 would need laser treatment for severe ROP (6, 11, 14). If 50% of them had access to appropriate management, 800 infants would be at risk of severe visual impairment throughout their lives every year. In 10 years, this number would rise to 8,000 visually impaired infants due to ROP.

The main obstacles for a comprehensive and effective ROP screening in Brazil, and probably in other middle-income countries, are the lack of skilled ophthalmologists to provide ROP care, the uneven distribution of these professionals among units, and the unequal access to appropriate quality neonatal care (11, 15–17). An alternative to improve accessibility and quality ROP care is the use of wide-field imaging (16, 17). Several authors reported good mean diagnostic accuracy for ROP type 2 or worse, feasibility, and cost-effectiveness of wide-filed imaging compared with screening with indirect binocular ophthalmoscopy (17–20).

However, wide-field imaging is not adopted for ROP screening in Brazil. The aim of this study was to perform a cost–utility analysis to compare two ROP screening strategies (indirect binocular ophthalmoscopy and combination of wide-field imaging with indirect binocular ophthalmoscopy) under the SUS's perspective. To the best of our knowledge, this is the first cost–utility analysis for ROP care, using the combination of wide-filed imaging and indirect ophthalmoscopy, from a governmental health system perspective of a middle-income country and with a long-term (lifetime) follow-up.

## Methods

### Setting and Population

Brazil, a middle-income country with a large universal governmental health system, has one of the highest numbers of infant survivors with severe visual impairment or blindness due to ROP (1, 21). The Brazilian guideline for ROP appropriate screening and timely treatment was published in 2007 (22). The governmental ROP screening care provides diagnosis and treatment at no cost to infants at risk for ROP. However, current ROP screening care has an estimate coverage of only 52% in Brazil (6, 11).

The eligible population included preterm infants with GA <32 weeks or BW ≤1,500 g born in governmental maternities in Brazil. Twenty-two governmental maternities from the city of Rio de Janeiro, Brazil, were used as a proxy to estimate the target population. The number of at-risk ROP infants (983) in these 22 maternities was estimated according to official (13). Non-treated and treated preterm infants needed a mean of 3 and 9 examinations, respectively, before discharge, resulting in a total of 3,254 annual examinations in Rio de Janeiro (11).

### Model Structure and Assumptions

**Figure 1:**
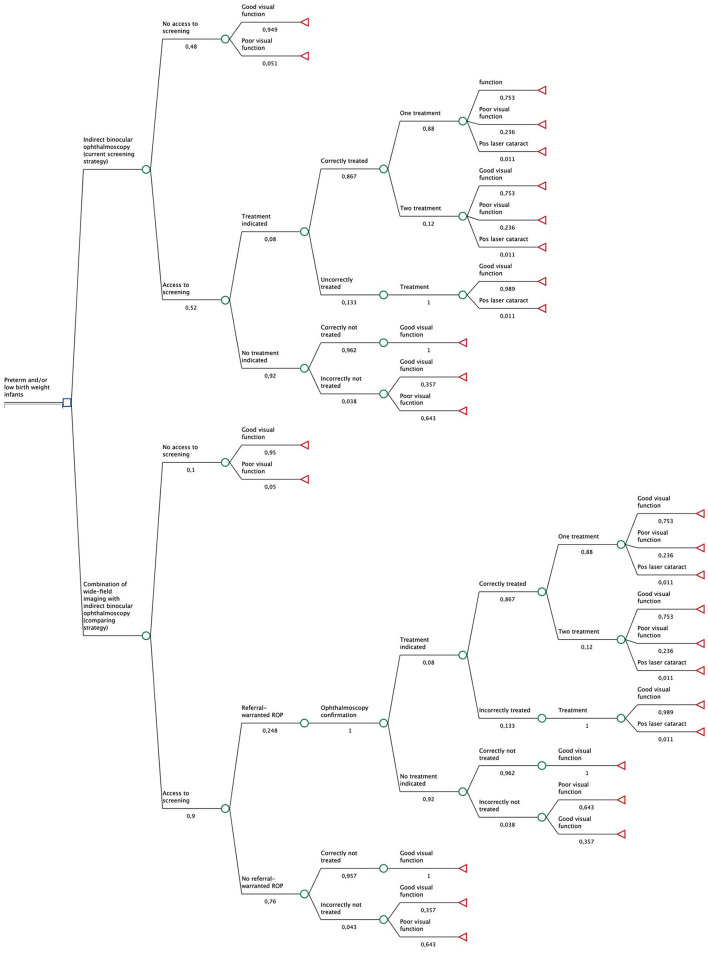
Decision analytic model for ROP care in preterm and/or low birth weight infants with two strategies: the current stratagy, indirect binocular ophthalmoscopy, and the comparing strategy, combination of wide-field imaging with indirect binocular ophthalmoscopy. The number under the branches of the decision tree represents the probability of each parameter branch to happen.

A decision analytical model for ROP care (screening, treatment, and long-term follow-up) was created simulating a cohort of preterm infants for a lifetime time horizon and from the SUS's perspective (Figure 1). The decision tree was used to represent the probability of the eligible population's possible health events and visual function outcomes in terms of cost and utility.

Three different visual acuities were defined: (1) good visual function in treated and non-treated infants was defined as 0.5 decimal visual acuity, because the eligible population usually has other morbidities that can affect the maximum potential visual acuity (1.0 decimal visual acuity). (2) Poor visual function in infants with severe ROP not treated was defined as 0.0 decimal visual acuity (23). (3) Poor visual function in treated infants with unfavorable outcome was defined as 0.05 decimal visual acuity (5).

The gold standard strategy is indirect binocular ophthalmoscopy performed by an ROP expert ophthalmologist. Eligible preterm infants can have access to gold standard ROP screening or not. Infants with no access to care can develop good or poor visual function, according to ROP's clinical course. Infants with access to care may need treatment or not. Infants submitted to one or two treatments may be correctly or incorrectly treated. They may develop good or poor visual function according to ROP's clinical course or, rarely, due to post-laser cataract. Infants not submitted to treatment can have severe ROP or not and therefore develop good or poor visual function.

The combined strategy considered the performance of wide-field imaging by non-ophthalmologists and remote reading by ROP expert ophthalmologist together with standard indirect binocular ophthalmoscopy of suspected (ROP type 2 or worse) or non-readable images. Eligible preterm infants can have access to the combined screening strategy or not. Infants with no access to care can develop good or poor visual function, according to ROP's clinical course. Infants with access to care are submitted to wide-field imaging. Suspected and unreadable images are referred to ophthalmoscopy and follow the aforementioned model. Infants with non-suspected images will not be referred. If this image reading is correct, they do not have ROP and will develop good visual function. However, if the image is misread and they have any ROP, they, incorrectly, will not be referred and can develop good or poor visual function, according to ROP's clinical course.

Assumptions were required to develop the screening model. All screened infants survived hospitalization. The treatment criteria followed the Early Treatment for Retinopathy of Pre-maturity Study (5), and all infants requiring treatment had bilateral laser treatment. Cataract was the only complication from laser treatment, and those infants developed low vision and intermediate utility. Non-readable images were referred to ophthalmoscopic examination. Final vision function in non-treated 15-year-old patients (23) and in treated 6-year-old patients (5) remained stable for life. All patients had an average life expectancy of 75 years. Anesthesia and additional hospitalization costs were not included.

The outcome was the incremental cost–utility ratio (ICUR), which was calculated as incremental cost per quality-adjusted life-years (QALY) gained per infant for ROP screening with the combined strategy compared with standard strategy, discounting future costs and QALYs at 5% per year (24).

TreeAge Pro 2011 was used to run the model (TreeAge Software, Inc., Williamstown, MA, USA).

### Model Parameters

#### Effectiveness Data

Sensitivity and specificity used for indirect binocular ophthalmoscopic diagnosis of ROP requiring treatment were, respectively, 0.867 and 0.962 (10, 25). Sensitivity and specificity for imaging reading of type 2 ROP or worse were, respectively, 0.768 and 0.957 (10, 25).

#### Utility Data

Utility valuations were based on decimal visual acuity following the formula: utility = 0.374. *x* + 0.514 (26), with *x* representing decimal visual acuity in better-seeing eye. Utility for good visual function in treated and non-treated infants was estimated as 0.701 (0.5 decimal visual acuity). Utility for poor visual function infants with severe ROP not treated was estimated as 0.514 (0.0 decimal visual acuity) (23), and utility for poor visual function in treated infants with unfavorable outcome was estimated as 0.5327 (0.05 decimal visual acuity) (5).

#### Cost Data

##### Wide-Field Imaging Cost

Wide-field imaging cost was calculated using a micro-costing approach as this procedure is not provided by SUS. The following items were considered: equipment kit (portable digital retinography, 130° lens, extra pedal, annual security contract, and maintenance), consumables (anesthetic and mydriatic eye drops, ophthalmic gel, lid speculum, gauze, glucose solution, antiseptic product, 20 diopter lens and gas), and staff (professional, training, traveling, and uploading images time). The equipment kit cost was based on market value provided by the manufacturer. The costs of consumables were obtained from official data (27). Costs were annualized using a standard discount rate of 5% (24) and an estimated equipment lifespan of 5 years and pedal lifespan of 2 years (6).

Staff costs were calculated by a time and motion study undertaken at two separate time points in order to assess the learning curve of nurse technicians trained by ophthalmologists. The first time point included 20 patients (40 eyes), and the second, 1 month later, 16 patients (32 eyes) (28). The second time point was considered in the calculation of staff costs. A mean of 10 min between each retinography was given to include dietary time or examination by other neonatal intensive care unit specialties. Training included equipment setup, image capturing, uploading, and equipment dismantling. Two ROP specialist ophthalmologists also had their mean time calculated for remote imaging reading (20 images). A mean of 5 min was given between each reading. Mean time spent per examination was used for estimating staff cost per examination and the number of professionals needed to cover the hypothetical screening program.

Wide-field imaging unit cost was calculated dividing the total cost of equipment, maintenance, consumables, and staff by the number of examinations.

##### Indirect Binocular Ophthalmoscopy and Treatment Costs

Indirect binocular ophthalmoscopy and treatment unit costs were based on published and updated data (6). The following items were considered: equipment kit (indirect binocular ophthalmoscopy, 20-diopter lens), consumables (anesthetic and mydriatic eye drops, lid speculum, depressor, gauze, glucose solution, and antiseptic product), and professional and training staff. Costs were annualized using a standard discount rate of 5% (24) and an estimated equipment life of 10 years (6).

##### Combination of Wide-Field Imaging and Indirect Binocular Ophthalmoscopy Cost

The combination's unit cost was estimated assuming 100% of infants would need wide-field imaging and 25.8% of them would be referred to ophthalmoscopy. Infants referred to ophthalmoscopy include 20.8% of ROP type 2 or worse and 5% of non-readable images (10).

##### Follow-Up Cost

Follow-up costs were split into three categories of eye care needs: (1) non-treated infants with good visual function; (2) treated infants with good visual function; (3) treated or non-treated infants with poor visual function. Costs were estimated according to an expert opinion based on frequency of ophthalmological and occupational therapy visits and on recommended complementary examinations (examination under anesthesia and Teller Visual Acuity Cards). The National Reimbursement Table underestimates true health procedures cost, and therefore, values were adjusted by 3.51 for follow-up estimated cost (29).

In 2019, costs were presented in Brazilian reais (R $) and then converted to US dollars (US $) (at the rate of US $1 = R $3.94 considering the mean rate from March to July 2019). Societal costs (indirect costs, loss of productivity, or cost of death) were not included.

### Sensibility Analysis

One-way sensitivity analysis was performed on all parameters of the model. Probabilities and utility ranges were obtained from literature. Costs ranged from −30% to + 30% (9) for ophthalmoscopy and treatment, from −30% to +60% for wide-field imaging, and from 0% to 10% discount rate for follow-up. The Monte Carlo probabilistic simulation, running 1,000 iterations samples, was performed to determine the probability of key parameters impacting the results. Beta, gamma, and lognormal probability distributions were applied for utilities, costs, and clinical parameters, respectively.

Brazil does not have a willingness-to-pay threshold to support healthcare technologies' adoption by SUS. A cost-effectiveness acceptability curve was constructed considering threshold values proposed for Brazil based on a range below US $7,544/QALY (30).

### Correlation Between QALY and Visual Acuity

The difference between normal (0.5) and abnormal (0) decimal visual acuity in the model was correlated with the difference between QALY in good (13.66) and poor visual function (10.01), respectively.

### Ethics Statement

The study was approved by the Ethical Review Board of Instituto Nacional de Saúde da Mulher, da Criança e do Adolescente Fernandes Figueira, Fundação Oswaldo Cruz, in Rio de Janeiro, Brazil.

## Results

The model parameters and range values are shown in Table 1. The ICUR of combined screening strategy (wide-field imaging in all at-risk ROP infants and indirect binocular ophthalmoscopy in referral cases) was US $1,746.99/QALY. Costs per examination by each screening strategy, treatment, and follow-up are shown in Table 1.

**Table 1:** Parameter estimates used in the model divided by screening, treatment, and follow-up categories.

**Parameters estimate**	**Baseline**	**Range (low–high)**	**Source**	**PSA**
**Screening**				
Access to ophthalmoscopy	0.52	0.20–0.80	(6) Assumption	Lognormal
Access to combination	0.90	0.52–0.95	Assumption	Lognormal
Ophthalmoscopy sensitivity	0.867	0.70–0.90	(10, 25), assumption	Lognormal
Ophthalmoscopy specificity	0.962	0.70–0.97	(10, 25), assumption	Lognormal
Wide-field imaging sensitivity	0.933	0.455–0.952	(10, 17)	Lognormal
Wide-field imaging specificity	0.962	0.617–0.98	(10, 17), Assumption	Lognormal
Good-quality imaging	0.95	0.90–0.98	(25), Assumption, (19)	Lognormal
ROP type 2 or worse	0.208	0.05–0.25	(10, 17, 37)	Lognormal
No. of examinations in infants not requiring laser treatment	(3)	1–13	Unit observation from 2006 to 2019	Lognormal
No. of examinations in infants requiring laser treatment	(9)	2–15	Unit observation from 2006 to 2019	Lognormal
**Treatment**				
ROP needing treatment	0.08	0.07–0.10	(6)	Lognormal
Two treatments needed	0,12	0.11–0.20	Unit observation from 2006 to 2019, (20), assumption	Lognormal
Facectomy (cataract surgery)	0.0109	0.005–0.05	(38), Assumption	Lognormal
Good visual function after treatment	0.753	0.60–0.857	(5), Assumption, (39)	Lognormal
Poor visual function when treatment is indicated and not performed	0.643	0.50–0.80	(23), assumption	Lognormal
**Utility/QALY estimates**				
Good visual function	0.701/13.65	7.0–66.6	(20, 26), discount rate 0–10%	Beta
Poor visual function				
Blindness	0.514/10.01	4.79–38.55	(20, 23, 26), discount rate 10–0%	Beta
Visual impairment	0.5327/10.38	5.32–39.95	(5, 26), discount rate 0–10%	Beta
**Costs estimate (US $)**				
Ophthalmoscopy	34.36	24.05–44.66	(6), −30% +30%	Gamma
Equipment	3.16		(27)	
Inputs	0.87		(27)	
Human resources (professional + training)	30.32 (23.43+6.89^*1^)		(6, 40, 41)	
Wide-field imaging	64.35	42.05–102.97	−30%, +60%	Gamma
Equipment	35.02		Market value	
Inputs	1.13		(27)	
Human resources (professional + training)	28.15 (28.04+0.11^*2^)		(40–44)	
Combination	58.20^*3^	40.74–93.12	−30%, +60%	Gamma
Laser treatment	642.09	449.46–834.72	(6), −30% +30%	Gamma
Equipment	502.92		(27)	
Inputs	0.87		(27)	
Human resources (professional + training)	134.26 (72.22+62.04^*4^)		(6, 40, 41)	
Cataract surgery (facectomy)	795.20	556.64–1,033.75	(45), −30% +30%	Gamma
**Follow-up**				
Good visual function in non-treated infants	69.33^*5^	44.17–230.96	(45), Discount rate range 10–0%	Gamma
Good visual function in treated infants	190.63^*5^	106.53–675.13	(45), Discount rate range 10–0%	Gamma
Poor visual function	286.91^*5^	195.04–781.16	(45), Discount rate range 10–0%	Gamma

*Parameters included clinical, epidemiological, utility and costs data and are presented as baseline and ranges values (high and low) with sources. Distribution of logarithmical probabilistic sensitivity (PSA) are presented for each parameter*.

*QALY, quality-adjusted life-years; ROP, retinopathy of pre-maturity; US $, US dollar*.

*1 *Ophthalmoscopy training 33 h^(6)^*.

*2 *Wide-field imaging training 500 min*.

*3 *100% wide-filed imaging and 25.8% ophthalmoscopy*.

*4 *Treatment training 33 h^(6)^*.

*5 *Discount rate 5%*.

Nurse technicians' learning curve to perform anterior and posterior retinography resulted in an efficiency gain of 45% (from 22 to 13 min per examination), and to set up and dismantle equipment, of 12% (from 13 to 12 min). The performance training lasted 500 min. In a 6-h shift, each team would be able to perform 10 to 13 examinations per day, that is, 250 to 325 examinations per month (150 h). Mean time spent for remote imaging reading by ROP specialists was 5 min per image. On average, 12 images were read per hour, that is, 1,200 images per month (100 h).

**Figure 2:**
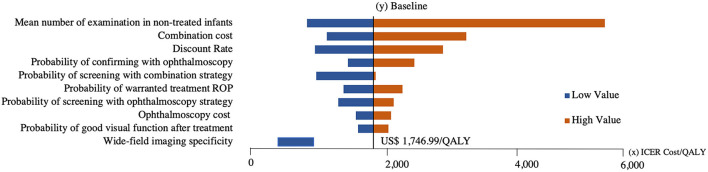
Tornado diagram of the 10 parameters that most impacted the incremental cost–utility ratio (ICUR). *X* axis represents the impact on the ICUR in US dollars (USD) cost per quality-adjusted life-years (QALY) of each parameter variation, and *Y* axis represents baseline parameters with an ICUR of USD 1,746,99/QALY. Orange and blue bands show each parameter high and low values, respectively.

**Figure 3:**
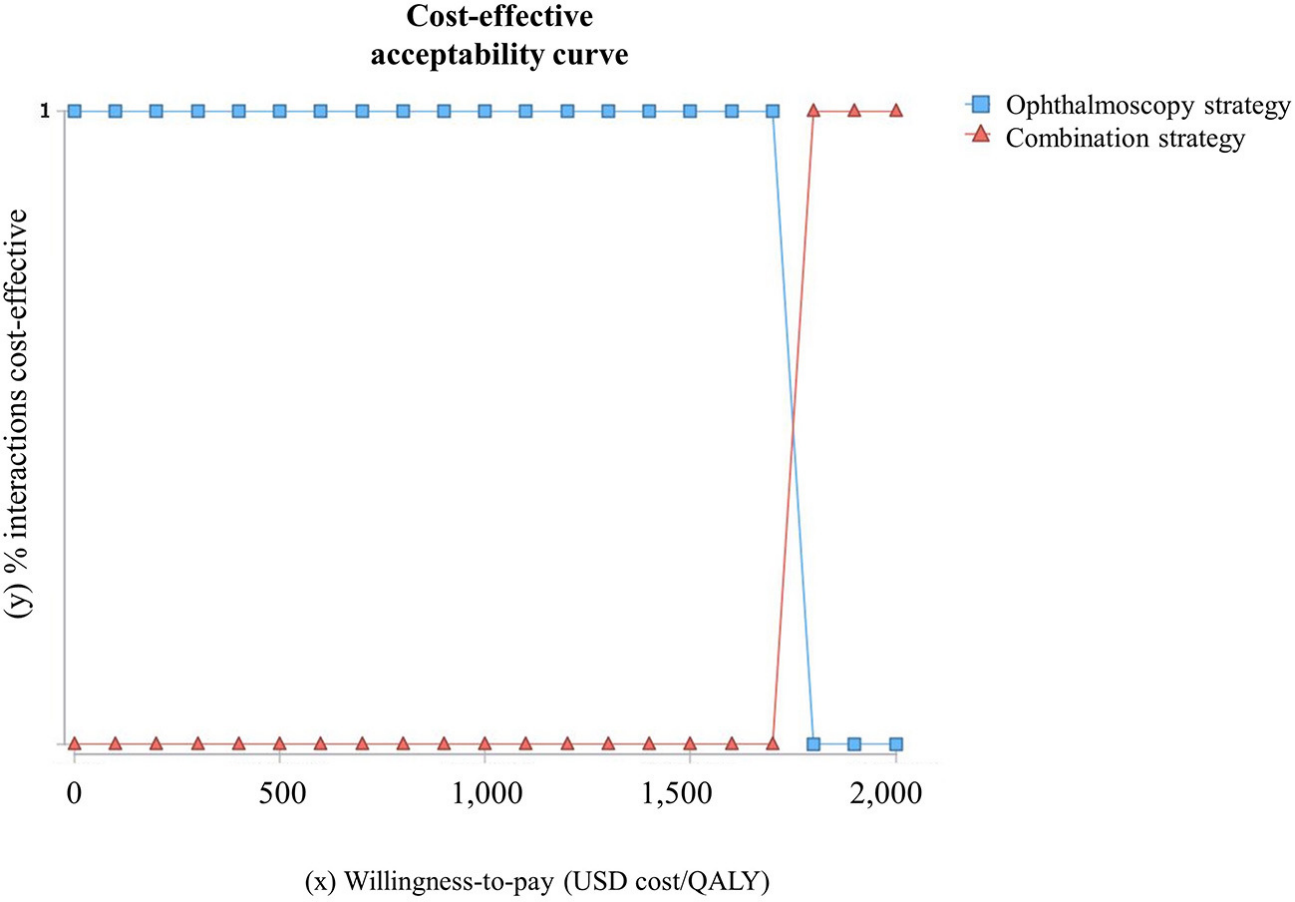
Monte Carlo probabilistic cost-effective acceptability curve. Interactions cost-effective percentages (*y*) between combination strategy (blue square) and ophthalmoscopy strategy (red triangle) by willingness-to-pay (WTP) (*x*) ranging from 0 to USD 2,000/QALY. Above USD 1,800/QALY combination strategy is 100% cost-effective.

The model showed that the probability of infants developing severe visual impairment due to ROP was 5% in ophthalmoscopy strategy and 3.2% in combined strategy. The tornado diagram demonstrates the impact of that variation in each parameter (Figure 2). The Monte Carlo probabilistic simulation yielded a 100% probability of combination strategy being cost-effective in a willingness-to-pay threshold of approximately US $1,800/QALY (Figure 3) (30).

A correlation was established between the gain in QALY and the gain in visual acuity. Every 1.2 QALY gained represented five lines gained in the decimal visual acuity chart (Table 2).

**Table 2:** Correlation between decimal visual acuity and quality-adjusted life-years (QALY) gained by infants with good and poor visual functions.

**Decimal visual acuity**	**QALY**	**Correlation of decimal visual acuity/QALY**
0	0	
0.00625	0.261	
0.0125	0.521	5 lines/1.2 QALY
0.025	0.782	
0.05	1.043	
0.06	1.304	
0.08	1.564	
0.1	1.825	5 lines/1.2 QALY
0.13	2.086	
0.16	2.346	
0.2	2.607	
0.25	2.868	
0.32	3.129	5 lines/1.2 QALY
0.4	3.389	
0.5	3.65	

## Discussion

Portable wide-field imaging has been available since early 2000, and as such, it is not a new screening technology (31). However, its high cost has been a barrier for its widespread use, especially in low- and middle-income countries. The results of this study suggest that the screening strategy combining wide-field imaging by nurses with indirect binocular ophthalmoscopy for ROP care (screening, treatment, and follow-up) costs approximately US $1,700/QALY gained when compared with standard indirect binocular ophthalmoscopy alone.

More importantly, the combination could enhance ROP screening coverage, particularly in marginalized areas that lack ROP experts. In our model, 5% of infants screened with the ophthalmoscopy strategy developed visual impairment, in contrast to 3.2% with the combination strategy, meaning a reduction in 36%. In Brazil, considering that there are 37,000 infants at risk of ROP, 76% of them depend on governmental maternities, 90% of them could have access to the combined strategy, and that the difference of developing visual impairment between the two strategies is 1.8%, ~455 visually impaired children due to ROP could be avoided every year in Brazil (37,000 × 0.76 × 0.9 × 0.018). In other words, we would need to screen 55 children to avoid 1 visually impaired child. If they have a mean life expectancy of 75 years, this could prevent 34,000 blind-years (blind children × life expectancy) (32).

The combined strategy could also enhance ROP screening efficiency by reducing the opportunity cost of ROP expert ophthalmologists. Opportunity cost is the cost of the second-best option or the not-chosen option. Currently, an experienced professional screens 100% of at-risk infants and treats <10%, but with the combination, he/she could examine 26% of infants at high risk of severe ROP and use almost 75% of the remaining time to teach and train younger professionals, perform other surgeries, and do scientific research (17, 20, 33).

In addition, eye examination documentation is precise and objective with wide-field imaging. It enables exchanging clinical cases by ROP experts and in-training ophthalmologists and favors medical liability management (25, 33). Besides, it could facilitate the family's understanding of the disease and enhance follow-up adherence (16). Documentation could also be an opportunity for creating an online eye examination database accessible to families and healthcare professionals involved in the infant's care follow-up. Eye examination documentation with indirect ophthalmoscopy is also possible, and its cost has been estimated (34). However, the learning curve of a non-ophthalmologist to perform indirect ophthalmoscopy should be longer as it is a diagnostic examination learned during medical residency in ophthalmology.

Previous economic modeling studies have included the cost-effectiveness of ROP screening, treatment, and follow-up (7–9, 20, 25, 34). Regarding wide-field imaging, one study of cost–utility analysis for ROP management adopted a third-party-payer perspective from the US Current Procedural Terminology with a 3-month post-operative follow-up (25), and another study adopted the UK National Health System's perspective with a 10-year follow-up (20). However, our study included a lifetime perspective and the cost of cataract surgery as a complication following laser treatment unlike these other authors (20, 25).

This study adopted three visual acuity levels to estimate three utility values and projected a very low incremental utility (0.044), similar to Jackson et al. (25) (0.0375). Using Castillo-Riquelme's utility values in the sensitivity analysis, which were higher for good visual function and lower for poor visual function than ours, there was also a low incremental utility value (0.088) (20). This low incremental utility reinforces the effectiveness of both screening strategies (ophthalmoscopy and combination). Therefore, wide-field imaging does not replace standard ophthalmoscopy, but it can have a crucial role in creating an ROP care program to enhance both: screening coverage and healthcare system efficiency.

Our group estimated that the gain of a single QALY represents an improvement of five lines in visual acuity chart. This estimate does have flaws due to the elasticity concept as the utility gained by a blind child is higher than the utility gained by a low vision patient. However, this estimate enables specialists who are not familiarized with the term “utility,” to better understand the enormous QALY gained in terms of lines of visual acuity with this screening combination.

Wide-field imaging for ROP is currently not available within SUS; therefore, a micro-costing approach was executed to estimate its cost. Regarding this, the study's major strengths were training non-ophthalmologists for bedside wide-field imaging, which resulted in an efficiency gain of 32%. Nurse technicians improved more in performing wide-field imaging (45% improvement), which is a more difficult step, than in setting up and dismantling the equipment (12%). The improvement was better in anterior segment imaging (73%) and good-quality imaging selection steps (44%). The efficiency level will increase as the learning curve was compared after only 1 month of learning.

Another major point was measuring reading time per image by ROP expert ophthalmologists. Measuring the time spent per examination for retinography and for image reading enabled us to estimate precisely wide-filed imaging cost and learning curve, which was not seen in other studies (20, 25, 34). Also, this measure allowed for planning a logistic and feasible ROP care program in Rio de Janeiro, as seen in other regions (19, 35).

Castillo-Riquelme et al. also performed a micro-costing approach to estimate the wide-field imaging cost in four scenarios (20). In the scenario like ours, where a specialized nurse visits the neonatal unit care for image capture and the ophthalmologist is the reader, the estimated cost per examination was 28 pounds. Our wide-field screening cost per examination was 35% higher than theirs [45.28 pounds (at the rate of 1 pound = R $5.60)]. This can probably be explained by difference in equipment and staff costs (20).

Our equipment cost per examination (24.64 pounds) represents 58% and staff cost (19.80 pounds) 40% of the total wide-field imaging cost. Conversely, in Castillo-Riquelme's study, the equipment cost represents 29% (8 pounds) and the staff cost 71% (20 pounds) (20). The Brazilian equipment cost per examination is three times the cost in the United Kingdom, according to our estimates. There may be two reasons to explain this: first, the exchange rate for acquiring the equipment in Brazil; second, the National Sanitary Vigilance Agency currently allows commercialization of only one equipment supplier. New suppliers could make the price more competitive.

The tornado diagram showed the parameters that most affected the ICUR were number of examinations in non-treated infants, combination strategy cost, and discount rate. The first can be explained because infants not treated represent 90% of all screened infants; therefore, the higher the number of their examinations during screening, the higher the total cost. However, the frequency of the upper limit of 10 examinations, in our experience, occurs in only 2% of the cases, whereas the frequencies of 2 and 3 examinations occur in 67 and 44%, respectively. The second parameter, combination strategy cost, also affected the model as when the cost is 60% higher, the ICUR doubles (US $3,849.15/QALY), and when the cost is 30% lower, the ICUR diminishes 60% (US $697.62/QALY). Moreover, the discount rate ranging from 0 to 10% also impacted the ICUR because the higher the discount rate, the lower the expected present value, especially in a lifetime time horizon.

Probabilistic analysis yielded a 100% likelihood of the combination strategy being cost-effective with a willingness-to-pay threshold of US $1,800. Brazil does not have a cost per QALY threshold for healthcare technology incorporation such as the United Kingdom, Canada, Chile, Colombia, or Mexico. The establishment of a threshold could facilitate healthcare technology evaluation interpretation and decision making (29).

Following the emergent concept of universal eye screening, where wide-field imaging is proposed to screen all term and preterm newborns (36), combination cost could be diminished because of the efficiency gain in screening all living infants with a low marginal cost. In other words, combination cost could be lower in a universal eye screening scenario, compared with an ROP screening scenario. Nevertheless, screening all living infants may raise concern regarding many unnecessary examinations performed in healthy infants or in infants with benign and transient alterations, such as retinal hemorrhage after vaginal delivery, which are the most common ocular alterations found (36).

Our study has some limitations that should be mentioned. First is the use of a mathematical formula to estimate the utility, for lack of better measurements, in visually impaired children. However, we found a similar low incremental utility (0.088) using more extreme utility values (high and low intervals) in the sensitivity analysis based on another study (20), which supports that the formula can be a reasonable and available option. Nevertheless, specific questionnaires for visual impairment in children are needed for a better utility estimate. Second, using assumptions in the model can cause some shortcomings in the results. However, we used assumptions for both strategies compared, minimizing these shortcomings. Third, costs of anesthesia and hospitalization, performed in both screening strategies, were not included, so the incremental cost should have the same magnitude. Fourth, costs for image printing were not included, although it is possible to create a sustainable program where images could be sent to a cellphone or by e-mail.

Some challenges remain for implementing the combined strategy. Because of the ever-developing nature of wide-field imaging, equipment leasing from the manufacturer, instead of its purchase, could result in a considerable benefit. This would allow not only a decrease in expense but also the adoption of more modern equipment as it becomes available. Another interesting challenge would be the implementation of a centralized imaging center that could receive, organize, and send the images to expert readers, as estimated by Mohammadi et al. (34). This center could even be responsible for training professionals and for the follow-up of infants after their discharge. In this scenario, other direct costs (infrastructure, staff, input) would have to be included.

More studies showing wide-field imaging accuracy for other eye diseases in infants could make it more efficient and facilitate its widespread use under the emergent concept of universal eye screening at birth. In addition, further studies focusing on the economic burden of this disease are also crucial to address the problem. Including indirect costs of visual impairment in childhood (as loss of productivity or governmental subsidies) and using the societal perspective could make the combination strategy even more affordable. Finally, our model has a long-life expectancy with low prospects of infants in need of treatment and of infants at risk of visual impairment. The use of another model considering the infant's coverage as the outcome could also enhance the analysis.

In conclusion, we have presented that the combination of wide-field imaging with binocular indirect ophthalmoscopy in specific referral cases is feasible and cost-effective for ROP screening from a middle-income country's perspective. It could also be a cost-saving strategy if the screening includes all living newborns. Moreover, the combination could enhance ROP screening coverage and health efficiency in middle-income countries, reducing the leading cause of childhood visual impairment in these countries.

## Data Availability Statement

The raw data supporting the conclusions of this article will be made available by the authors, without undue reservation.

## Ethics Statement

The studies involving human participants were reviewed and approved by Instituto Nacional de Saúde da Mulher, da Criança e do Adolescente Fernandes Figueira, Fundação Oswaldo Cruz, Rio de Janeiro, RJ, Brazil. The patients/participants provided their written informed consent to participate in this study.

## Author Contributions

AAZ, ZFMV, and MP: conceptualization. LMN, LMH, AAZ, and MP: methodology. LMN, LMH, AAZ, and MP: validation. LMN, LMH, AAZ, RES, ZFMV, and MP: formal analysis. LMN, LMH, AAZ, and MP: resources. LMN, LM, AAZ, and MP: data curation. LMN, LMH, AAZ, and MP: writing original draft and preparation. LMN, LMH, AAZ, RES, ZFMV, and MP: writing, review, and editing. AAZ and MP: supervision. AAZ and MP: project administration. All authors have read and agreed to the published version of the manuscript.

## Conflict of Interest

The authors declare that the research was conducted in the absence of any commercial or financial relationships that could be construed as a potential conflict of interest.

## Publisher's Note

All claims expressed in this article are solely those of the authors and do not necessarily represent those of their affiliated organizations, or those of the publisher, the editors and the reviewers. Any product that may be evaluated in this article, or claim that may be made by its manufacturer, is not guaranteed or endorsed by the publisher.

## References

[1] Blencowe H Lawn JE Vazquez T Fielder A Gilbert C. Preterm-associated visual impairment and estimates of retinopathy of prematurity at regional and global levels for 2010. Pediatr Res. (2013) 74(Suppl. 1):35–49. 10.1038/pr.2013.205 24366462 PMC3873709

[2] Kong L Fry M Al-Samarraie M Gilbert C Steinkuller PG. An update on progress and the changing epidemiology of causes of childhood blindness worldwide. J AAPOS. (2012) 16:501–7. 10.1016/j.jaapos.2012.09.004 23237744

[3] Quinn GE. Retinopathy of prematurity blindness worldwide: phenotypes in the third epidemic. Eye Brain. (2016) 8:31–6. 10.2147/EB.S94436 28539799 PMC5398741

[4] Eds Howson CP Kinney MV Lawn JE. March of Dimes, PMNCH, Save the Children, WHO. Born Too Soon: The Global Action Report on Preterm Birth. Geneva: World Health Organization (2012).

[5] Early Treatment for Retinopathy of Prematurity Cooperative Group Good WV Hardy RJ Dobson V Palmer EA Phelps DL. Final visual acuity results in the early treatment for retinopathy of prematurity study. Arch Ophthalmol. (2010) 128:663–71. 10.1001/archophthalmol.2010.72 20385926 PMC4162423

[6] Zin AA Magluta C Pinto MFT Entringer A Mendes-Gomes MA Moreira ME. Retinopathy of prematurity screening and treatment cost in Brazil. Rev Panam Salud Publica. (2014) 36:37–43. 25211676

[7] Brown GC Brown MM Sharma S Tasnam W Brown HC. Cost-effectiveness of treatment for threshold retinopathy of prematurity. Pediatrics. (1999) 104:e47. 10.1542/peds.104.4.e47 10506272

[8] Dunbar JA Hsu V Christensen M Black B Williams P Beauchamp G. Cost-utility analysis of screening and laser treatment of retinopathy of prematurity. J AAPOS. (2009) 13:186–90. 10.1016/j.jaapos.2008.10.014 19393519

[9] Rothschild MI Russ R Brennan KA Williams CJ Berrones D Patel B. The Economic Model of Retinopathy of Prematurity (EcROP) screening and treatment: Mexico and the United States. Am J Ophthalmol. (2016) 168:110–21. 10.1016/j.ajo.2016.04.014 27130372

[10] Chiang MF Starren J Du YE Keenan JD Schiff WM Barile GR. Remote image based retinopathy of prematurity diagnosis: a receiver operating characteristic analysis of accuracy. Br J Ophthalmol. (2006) 90:1292–6. 10.1136/bjo.2006.091900 16613919 PMC1857452

[11] Zin AA Moreira ME Bunce C Darlow BA Gilbert CE. Retinopathy of prematurity in 7 neonatal units in Rio de Janeiro: screening criteria and workload implications. Pediatrics. (2010) 126:e410–7. 10.1542/peds.2010-0090 20660549

[12] REDE Interagencial de Informação para a Saúde. Indicadores Básicos para a Saúde no Brasil: Conceitos e Aplicações / Rede Interagencial de Informação para a Saúde – Ripsa. Brasília: Organização Pan-Americana da Saúde (2008).

[13] DATASUS Informações de Saúde, Estatísticas, Vitais. (2019). Available online at: http://www2.datasus.gov.br/DATASUS/index.php?area=0205 (acessed March 15, 2019).

[14] Guinsburg R de Almeida MF de Castro JS Silveira RC Caldas JP Fiori HH. Death or survival with major morbidity in VLBW infants born at Brazilian neonatal research network centers. J Matern Fetal Neonatal Med. (2016) 29:1005–9. 10.3109/14767058.2015.1031740 25812674

[15] Costa MdFdS Gomes Junior SC Magluta C. Análise da distribuição dos nascimentos com marcadores de gravidade em maternidades com unidade de terapia intensiva neonatal no Sistema Único de Saúde. Cadernos Saúde Coletiva. (2018) 26:125–30. 10.1590/1414-462x201800020419

[16] Quinn GE Vinekar A. The role of retinal photography and telemedicine in ROP screening. Semin Perinatol. (2019) 43:367–74. 10.1053/j.semperi.2019.05.010 31153621

[17] Skalet AH Quinn GE Ying GS Gordillo L Dodobara L Cocker K. Telemedicine screening for retinopathy of prematurity in developing countries using digital retinal images: a feasibility project. J AAPOS. (2008) 12:252–8. 10.1016/j.jaapos.2007.11.009 18289897

[18] Fierson WM Capone A Jr American Academy of Pediatrics Section on Ophthalmology, American Academy of Ophthalmology, American Association of Certified Orthoptits. Telemedicine for evaluation of retinopathy of prematurity. Pediatrics. (2015) 135:e238–54. 10.1542/peds.2014-0978 25548330

[19] Ossandon D Zanolli M Stevenson R Agurto R Ortiz P Dotan G. A national telemedicine network for retinopathy of prematurity screening. J AAPOS. (2018) 22:124–7. 10.1016/j.jaapos.2017.11.005 29535051

[20] Castillo-Riquelme MC Lord J Moseley MJ Fielder AR Haines L. Cost-effectiveness of digital photographic screening for retinopathy of prematurity in the United Kingdom. Int J Technol Assess Health Care. (2004) 20:201–13. 10.1017/S0266462304000984 15209180

[21] Furtado JM Lansingh VC Carter MJ Milanese MF Pena BN Ghersi HA. Causes of blindness and visual impairment in Latin America. Surv Ophthalmol. (2012) 57:149–77. 10.1016/j.survophthal.2011.07.002 22137039

[22] Zin AA Fortes filho JB Nakanami CR Gianini N Graziano RM Moraes N. Proposta de diretrizes brasileiras do exame e tratamento de retinopatia da prematuridade (ROP). Arq Bras Oftalmol. (2007) 70:875–83. 10.1590/S0004-27492007000500028 18157319

[23] Palmer EA Phelps DL Quinn GE Summers CG Krom CP Tung B. Cryotherapy for Retinopathy of Prematurity Cooperative Group. 15-year outcomes following threshold retinopathy of prematurity: final results from the multicenter trial of cryotherapy for retinopathy of prematurity. Arch Ophthalmol. (2005) 123:311–8. 10.1001/archopht.123.3.311 15767472

[24] Ministério da Saúde. Secretaria de Ciência, Tecnologia e Insumos Estratégicos. Departamento de Ciência e Tecnologia. Diretrizes metodológicas: Diretriz de Avaliação Econmica. Brasília: Ministério da Saúde (2014).

[25] Jackson KM Scott KE Zivin JG Bateman DA Flynn JT Keenan JD. Cost-utility analysis of telemedicine and ophthalmoscopy for retinopathy prematurity management. Arch Ophthalmol. (2008) 126:493–9. 10.1001/archopht.126.4.493 18413518 PMC12309845

[26] Sharma S Brown GC Brown MM Shah GK Snow K Brownt H. Converting visual acuity to utilities. Can J Ophthalmol. (2000) 35:267–72. 10.1016/S0008-4182(00)80077-0 10959467

[27] Ministério da Economia. Painel de Preços (2019). Available online at: https://paineldeprecos.planejamento.gov.br/ (acessed March 15, 2019).

[28] Pinto M Entringer AP Steffen R Trajman A. Cost analysis of nucleic acid amplification for diagnosing pulmonary tuberculosis, within the context of the Brazilian Unified Health Care System. J Bras Pneumol. (2015) 41:536–8. 10.1590/s1806-37562015000004524 26785963 PMC4722795

[29] Pinto M Steffen RE Cobelens F van den Hof S Entringer A Trajman A. Cost-effectiveness of the Xpert(R) MTB/RIF assay for tuberculosis diagnosis in Brazil. Int J Tuberc Lung Dis. (2016) 20:611–8. 10.5588/ijtld.15.0455 27084814

[30] Woods B Revill P Sculpher M Claxton K. Country-level cost-effectiveness thresholds: initial estimates and the need for further research. Value Health. (2016) 19: 929–35. 10.1016/j.jval.2016.02.017 27987642 PMC5193154

[31] Schwartz SD Harrison SA Ferrone PJ Trese MT. Telemedical evaluation and management of retinopathy of prematurity using a fiberoptic digital fundus camera. Ophthalmology. (2000) 107:25–8. 10.1016/S0161-6420(99)00003-2 10647714

[32] Gilbert C Foster A. Childhood blindness in the context of VISION 2020 - the right to sight. Bull World Health Organ. (2001) 79:227–32. 11285667 PMC2566382

[33] Patel SN Martinez-Castellanos MA Berrones-Medina D Swan R Ryan MC Jonas KE. Assessment of a tele-education system to enhance retinopathy of prematurity training by international ophthalmologists-in-training in Mexico. Ophthalmology. (2017) 124:953–61. 10.1016/j.ophtha.2017.02.014 28385303 PMC7895299

[34] Mohammadi SF Rahban A Darabeigi S Salimi N Farahani A Lashay A. Cost-effectiveness analysis of tele-retinopathy of prematurity screening in Iran. Int J Ophthalmol. (2021) 14:560–6. 10.18240/ijo.2021.04.13 33875948 PMC8025162

[35] Vinekar A Mangalesh S Jayadev C Gilbert C Dogra M Shetty B. Impact of expansion of telemedicine screening for retinopathy of prematurity in India. Indian J Ophthalmol. (2017) 65:390–5. 10.4103/ijo.IJO_211_17 28573995 PMC5565892

[36] Vinekar A Govindaraj I Jayadev C Kumar AK Sharma P Mangalesh S. Universal ocular screening of 1021 term infants using wide-field digital imaging in a single public hospital in India - a pilot study. Acta Ophthalmol. (2015) 93:e372–6. 10.1111/aos.12685 25721891

[37] Wu C Petersen RA Van derVeen DK. RetCam imaging for retinopathy of prematurity screening. J AAPOS. (2006) 10:107–11. 10.1016/j.jaapos.2005.11.019 16678743

[38] Davitt BV Christiansen SP Hardy RJ Tung B Good WV Early Treatment for Retinopathy of Prematurity Cooperative Group. Incidence of cataract development by 6 months' corrected age in the Early Treatment for Retinopathy of Prematurity study. J AAPOS. (2013) 17:49–53. 10.1016/j.jaapos.2012.10.011 23352719 PMC3577978

[39] Good W. Early treatment for retinopathy of prematurity cooperative group. Final results of the Early Treatment for Retinopathy of Prematurity (ETROP) randomized trial. Trans Am Ophthalmol Soc. (2004) 102:233–48. 15747762 PMC1280104

[40] Divisão de Gestão Administrativa da Coordenação Geral do Núcleo Estadual do Ministério da Saúde no Rio de Janeiro. N° 01/2018. (2018). Available online at: http://www.nerj.rj.saude.gov.br/ (accessed July 10, 2019).

[41] Tabela de Remuneração dos Servidores Públicos Federais Civis e dos Ex-territórios. Ministério da Economia (2019). Available online at: http://www.planejamento.gov.br/assuntos/gestao-publica/arquivos-e-publicacoes/tabela-de-remuneracao-78-jan2019.pdf (accessed July 10, 2019).

[42] Prefeitura da Cidade do Rio de Janeiro. Secretaria Municipal da Casa Civil. Subsecretaria de Serviços Compartilhados. Edital Cvl/Subsc N° 20 de 28 de Janeiro de 2019 (2019). Available online at: http://www.rio.rj.gov.br/dlstatic/10112/9078371/4229016/editalenfermeiro_tecenfermagem_auxenfermagem2019revisadook.pdf (accessed July 10, 2019).

[43] Prefeitura da Cidade de Cabo Frio. Secretaria Municipal de Administração. Edital N° 01/2018 – Processo Seletivo Simplificado (2018). Available online at: http://processoseletivo.cabofrio.rj.gov.br/Edital-Secad-01-2018.pdf (accessed July 10, 2019).

[44] Fundação para o Desenvolvimento Científico e Tecnológico em Saúde. Chamada n° 07/2018 - Processo Seletivo Simplificado para Motorista (2018) Rio de Janeiro. Country: Brazil. Fundação para o Desenvolvimento Científico e Tecnológico em Saúde (Fiotec). (accessed July 10, 2019).

[45] Tabela de Procedimentos, Medicamentos Órteses Próteses e Materiais Especiais do Sistema Único de Saúde, (SIGTAP). (2020). Available online at: http://sigtap.datasus.gov.br/tabela-unificada/app/sec/inicio.jsp (accessed February 10, 2020).

